# Editorial: Imaging in non-small cell lung cancer

**DOI:** 10.3389/fonc.2023.1143204

**Published:** 2023-02-07

**Authors:** Yiyan Liu

**Affiliations:** Department of Radiology, University of Louisville School of Medicine, Louisville, KY, United States

**Keywords:** non-small cell lung cancer (NSCLC), radiomics, CT, PET/CT, NSCLC

Non-Small Cell Lung Cancer (NSCLC) accounts for approximately 80% to 85% of all lung cancer cases and is the leading cause of cancer death worldwide (1, 2). Beyond invasive histopathological diagnosis, imaging plays critical roles in screening, diagnosing, staging, restaging, detecting recurrence and/or metastasis, and monitoring therapeutic response of NSCLC. Significant limitations of traditional image modalities such as radiography, CT, and MRI have prompted the development of novel imaging strategies for better and more accurate tumor characterization and guidance for targeted therapy. Our Research Topic aimed to shed light on the latest imaging advances in NSCLC, which has been very attractive to our contributors and readers. We would like to express great appreciation for valuable contributions to the Research Topic by all authors.

Over the last two decades, F18-fluorodeoxyglucose PET/CT has been widely used for NSCLC, which has the unique advantage of metabolic evaluations with semi-quantitative parameters compared to the traditional images. Although there has been significant progress in FDG PET/CT applications and performance, further advancements can optimize imaging interventions as well as establish emerging approaches, such as radiomics, to improve the management of lung cancer. 8 of 17 published articles in our Research Topic are about the value of radiomics features in NSCLC.

Radiomics has been considered an “imaging biomarker”, and it is a quantitative approach with advanced mathematical analysis to medical imaging, which extracts many features from medical images using data-characterization algorithms. By providing 3-D characterization of the tumor, radiomics features based on CT, MRI and/or PET describe intra-tumor heterogeneity and have the potential to uncover disease characteristics. To date, radiomics has been most extensively studied in NSCLC compared to other tumors (3, 4). Published articles in the Research Topic have demonstrated new information about the values of radiomics in NSCLC.

Adequate identification of genotype and gene mutation is the basis for target therapy. Recent research showed correlation between the radiomics features and the epithelial growth factor receptor (EGFR) (5–7). In this topic collection, Zhang et al. reported that with correction of FDG PET/CT radiomics features to EGFR mutation analyses of tissue samples, FDG PET/CT radiomics models could help in discriminating EGFR positive from negative mutations in 173 preoperative patients with NSCLC, which could guide target therapy in patients with EGFR mutations. In another larger multicenter study including 728 patients with lung adenocarcinoma, Zhang et al. reported a two-user friendly nomogram by calculating radiomics score to predict the EGFR mutation status. Wu et al. also demonstrated that pre-treatment CT based radiomics features could reliably predict EGFR mutation in 67 NSCLC patients, and the addition of clinical models further improved radiomics performance.

Beyond invasive approach to histopathologic diagnosis by surgery or biopsy, radiomics may help in the prediction of histology and stage of the tumors. Tang et al. analyzed FDG PET/MRI radiomics in 61 NSCLC patients and found that FDG PET/MRI radiomics features revealed different degrees of correlation with different tumors and could predict the preoperative histological classifications of the lesions: adenocarcinoma or squamous cell cancer.

Differentiation between lung metastasis and the 2^nd^ lung primary is of incremental significance of treatment and prognosis in oncologic patients. Zhong et al. evaluated the value of CT radiomics in discriminating the second lung primary from lung metastasis in 252 oncologic patients with suspected lung lesions. 16 radiomics features and 4 clinical-radiographic features were selected to build the final model, which showed good discriminative capacity for the 2^nd^ lung primary and lung metastasis.

Currently immunotherapy by immune checkpoint blockade is standard care in advanced NSCLC. Radiomics has been used in assessment of PD-L1 expression in NSCLC (8). Zhou et al. assessed the predictive role of FDG PET/CT based radiomics for tumor microenvironment immune types (TMIT) in 103 treatment-naïve NSCLC patients, including the expressions of programmed death ligand (PD-L1), programmed death 1 (PD-1) and CDF8+ tumor infiltrating lymphocytes. The results suggested that FDG PET/CT radiomics features had good performance in predicting the TMIT.

Zha et al. developed and validated a nomogram model based on CT radiomics features for preoperative prediction of visceral pleural invasion in 659 patients with lung adenocarcinoma. The results showed that the nomogram combining clinical and radiomics features had markedly improved accuracy, specificity, positive predictive value, and AUC for predicting visceral pleural invasion.

The micropapillary pattern is a marker of poor prognosis in NSCLC (Xu et al.). Li et al. reported a radiomics model based on nodule type stratification for preoperative prediction of micropapillary pattern in lung adenocarcinoma less than 2 cm. They found that ground glass opacity nodule type affected performance of the prediction.

In conclusion, radiomics is expected to optimize and augment image capabilities, and holds the great promise of valuable clinical applications in diagnosis, staging, prediction of treatment outcome and survival in NSCLC. Today we are on the brink of a new era in radiology artificial intelligence (AI). AI and deep learning models will facilitate faster clinical translation and implementation of radiomics in NSCLC. However, the significant variability in radiomics features used in different studies, in addition to the lack of reproducibility, suggests that the current data are still preliminary and more comprehensive studies are needed for validation of radiomics applications.

## Author contributions

The author confirms being the sole contributor of this work and has approved it for publication.
